# Complete Genome Sequence of Carp Edema Virus Isolated from Koi Carp

**DOI:** 10.1128/MRA.00239-21

**Published:** 2021-04-22

**Authors:** Tohru Mekata, Yasuhiko Kawato, Takafumi Ito

**Affiliations:** a Pathology Division, Nansei Field Station, Fisheries Technology Institute, Japan Fisheries Research and Education Agency, Mie, Japan; Queens College CUNY

## Abstract

Carp edema virus disease (CEVD), or koi sleepy disease, is caused by CEV. Here, we report the complete genome sequence of CEV strain FTI2020, isolated from koi carp. This sequence information has great potential for improving our understanding of the genetic characteristics of this piscine poxvirus.

## ANNOUNCEMENT

The koi carp (Cyprinus carpio) is one of the most popular ornamental fish species, especially in Asia and several European countries. Disease outbreaks due to carp edema virus (CEV) were reported for the first time in the mid-1970s on Japanese koi farms (1, 2). It is currently one of the most serious pathogens in farmed carp worldwide (3). CEV, belonging to the *Poxviridae* family, has a double-stranded linear DNA genome sequence, and mature virions are ovoid to spherical in shape (4).

Naive carp were infected by immersion in a water bath with the gill homogenate of CEV-infected fish (collected from a koi farm in Niigata, Japan, in 2019). Virions were concentrated from the gills and fins using the sucrose gradient centrifugation method (5). Crudely purified viral particles were preserved in DNA/RNA Shield, and genomic DNA was extracted using a Quick-DNA high-molecular-weight (HMW) MagBead kit (both Zymo Research). Due to the limited amount of DNA available, the whole-genome sequence was amplified using the Illustra GenomiPhi v2 kit (Cytiva). The DNA quality was checked on a Qubit v4 fluorometer (Thermo Fisher Scientific) using a Qubit double-stranded DNA (dsDNA) broad-range (BR) assay kit (Invitrogen). Sequencing libraries were prepared using a rapid sequencing kit (Oxford Nanopore Technologies) and sequenced on an R9.4 flow cell with a MinION instrument (Oxford Nanopore Technologies). MinKNOW v19.12.5 software was used in FAST mode for base calling. Default parameters were used for all software unless otherwise specified. In total, 344,000 reads with an average length of 1,577 bp were obtained, and assembly was performed using CLC Genomics Workbench v21.0.3. A local database for a BLAST search was created using long-read sequences, and a full-length genome sequence was manually constructed by *in silico* genomic walking using CLC Genomics Workbench. Several uncertain regions remained in the draft genome sequence, and hence, genome polishing was performed with a higher-purity viral genome prepared from virions shed from CEV-infected fish. Briefly, the rearing water of CEV-infected fish was filtered through a 0.4-μm polycarbonate filter (Advantec) and concentrated using the iron flocculation method (6) after dissolving 1% artificial seawater compound (Osaka Yakken). DNA was directly extracted from the flocculate-trapped filter using the DNeasy blood and tissue kit (Qiagen). Library preparation using an MGI Easy FS DNA library prep set and 200-bp paired-end sequencing using the DNBSEQ-G400 platform (MGI Tech) was outsourced to Bioengineering Lab. Co., Ltd. (Japan). A total of 187,344 reads were mapped to the draft genome sequence, providing 80× average coverage, and were polished using CLC Genomics Workbench. Gene annotation was performed using OmicsBox v1.4.11 (Qiagen) (7).

The full-length genome sequence comprised 456,821 bp with a G+C content of 28.8%, and 392 genes were estimated. The inverted terminal repeats were located at both ends of the genome. Phylogenetic analysis using the MEGA X v10.1.8 program (8) showed that the CEV strain FTI2020 was classified as genogroup IIa based on the P4a core protein gene (9). The putative major capsid protein exhibited 36.7% amino acid identity with that of salmon gill poxvirus (GenBank Protein accession number QBX90040) by BLASTp analysis. This study was approved by the Institutional Animal Care and Use Committee of the National Research Institute of Aquaculture (permission number IACUC-NRIA 20007).

### Data availability.

The complete genome sequence and raw reads have been deposited in DDBJ/ENA/GenBank under the accession numbers LC613089, DRX267323, and DRX267324.
